# Food environment does not predict self-reported SSB consumption in New York City: A cross sectional study

**DOI:** 10.1371/journal.pone.0196689

**Published:** 2018-10-24

**Authors:** Ben R. Spoer, Jonathan H. Cantor, Pasquale E. Rummo, Brian D. Elbel

**Affiliations:** 1 Department of Social-Behavioral Studies, College of Global Public Health, New York University, New York City, New York, United States of America; 2 Department of Population Health, New York University School of Medicine, New York City, New York, United States of America; 3 Wagner School of Public Service, New York University, New York City, New York, United States of America; State University of Rio de janeiro, BRAZIL

## Abstract

The purpose of this research was to examine whether the local food environment, specifically the distance to the nearest sugar sweetened beverage (SSB) vendor, a measure of SSB availability and accessibility, was correlated with the likelihood of self-reported SSB consumption among a sample of fast food consumers. As part of a broader SSB behavior study in 2013–2014, respondents were surveyed outside of major chain fast food restaurants in New York City (NYC). Respondents were asked for the intersection closest to their home and how frequently they consume SSBs. Comprehensive, administrative food outlet databases were used to geo-locate the SSB vendor closest to the respondents’ home intersections. We then used a logistic regression model to estimate the association between the distance to the nearest SSB vendor (overall and by type) and the likelihood of daily SSB consumption. Our results show that proximity to the nearest SSB vendor was not statistically significantly associated with the likelihood of daily SSB consumption, regardless of type of vendor. Our results are robust to alternative model specifications, including replacing the linear minimum distance measure with count of the total number of SSB vendors or presence of a SSB vendor within a buffer around respondents’ home intersections. We conclude that there is not a strong relationship between proximity to nearest SSB vendor, or proximity to a specific type of SSB vendor, and frequency of self-reported SSB consumption among fast food consumers in NYC. This suggests that policymakers focus on alternative strategies to curtail SSB consumption, such as improving the within-store food environment or taxing SSBs.

## Introduction

According to data from the NYC Health and Nutrition Examination Survey, approximately one-third of New York City (NYC) adults were obese in 2013–14 [1]. Frequent consumption of sugar sweetened beverages (SSBs) has been associated with elevated risk of obesity.[2] In 2013, 23.3% of NYC adults reported consuming a SSB at least daily[3,4] and adult SSB drinkers consumed 193 calories from SSBs daily.[5] Thus, one way to lower obesity prevalence is to reduce the consumption of SSBs.

Local food environments are also associated with obesity[6], potentially via variation in access to healthy (nutrient-rich, low energy density) and unhealthy (nutrient-poor, energy dense) foods and beverages, among other factors.[7] Access to healthy or unhealthy foods and beverages is driven in part by differential access to food outlets.[7] For example, closer proximity to less healthy restaurants (e.g. fast food) and food store locations (e.g. corner stores or supermarkets) may increase unhealthy food and beverage consumption by increasing the availability of unhealthy foods and beverages.

With this in mind, this research is concerned with two facets of the local food environment, SSB accessibility and availability, as articulated in Caspi et. al. (2012).[7] Previous work suggests high SSB accessibility and availability in NYC. Corner stores and fast food restaurants represent a disproportionate percentage of the NYC food environment, and SSBs comprise a substantial proportion of overall product purchases in corner stores in NYC.[8] This puts NYC residents at an increased risk of high SSB consumption.

Few studies have examined the relationship between the local food environment and adult SSB consumption.[9–11] In Los Angeles, one study found no relationship between SSB intake and the distance between households and specific food outlet types.[11] However, no previous studies have explored how the distance from participants’ home intersection to the nearest food outlet is related to SSB consumption, nor does prior research focus on an area with ample active and public transportation options, like NYC.

To address these gaps, we used cross-sectional data on frequency of self-reported SSB consumption, spatially linked to comprehensive administrative neighborhood food resource data, to examine the associations between proximity to the nearest SSB vendor from home (overall and by vendor type) and self-reported SSB consumption frequency.

## Methods

Study staff collected receipts and surveys from customers exiting chain fast food restaurants in NYC during three rounds of data collection (January-April 2013, August-November 2013 and January-June 2014). The five fast food chains that had the most locations in NYC, as well as locations in New Jersey (which were used as a comparison group for a separate project) were surveyed on weekdays during lunch (11:30am-2:30pm) and dinner hours (4:30pm-7:30pm). Fast food chains were selected because they would best demonstrate the effect of the now defunct NYC Soda Portion Cap Rule, which the data collection approach was designed to examine. Customers were offered a $2 incentive to provide their receipt and fill out a survey. Participants were eligible for the study if they were at least 18 years old, had a receipt from the restaurant in question, and could communicate in English or Spanish. All participants provided verbal informed consent to participate. The study protocols were approved by the New York University Medical Center Institutional Review Board. The full sample included over 6,000 participants, which provided over 90% power for the original study. The subsample analyzed in this research included 3,266 participants, and provides over 90% power to detect a 50% change in the odds of consuming an SSB at least once a day.[11]

Respondents were surveyed about demographic characteristics (gender, age, race/ethnicity, education, and employment status), the intersection nearest their home, and their SSB purchase and consumption. Specifically, respondents were asked how many times in the past seven days they consumed a bottle or glass of regular (non-diet) soda, using a question adapted from the National Health and Nutrition Examination Survey. In addition, data collectors asked respondents “where do you buy regular soda most often?” Possible choices included supermarkets, fast food restaurants and corner stores.

Based on the response to the SSB frequency measure, we created a dichotomous measure for daily SSB consumption. To examine possible heterogeneity by type of restaurant or food store, we created separate dichotomous measures (yes/no) for purchasing an SSB at least daily from each type of SSB vendor, including fast food restaurants, wait service restaurants, bodegas, or supermarkets.

Using 2013–2014 licensing and inspection data from the New York State Department of Agriculture and Markets, we defined corner stores as food vendors less than 2,000 square feet (185.8 square meters) in area. Supermarkets were defined as locations greater than 5,000 square feet (464.5 square meters). Information on fast food restaurant locations was obtained from the NYC Department of Health and Mental Hygiene Restaurant Grading data, via annual inspections, also in 2013–2014. We attempted to geocode the home intersections for all NYC-based survey respondents, with a 56% success rate. We then calculated the linear distance from respondents’ home intersections to the nearest food outlet location using ArcGIS version 10.4. We did the same for each type of food outlet. We assumed that each food outlet sold SSBs.

We used logistic regression models to examine the associations between the distance to nearest food outlet location and (1) daily consumption (yes/no) of an SSB overall; and (2) daily consumption (yes/no) of an SSB by food outlet type (i.e., corner store, fast food restaurant, or supermarket). We controlled for the respondent’s age, gender, race/ethnicity (using dummies for non-Hispanic white, non-Hispanic African American, Hispanic, and non-Hispanic other race), education level, employment status, frequency of fast food restaurant visits in the past week, the borough in which their home intersection was located, the restaurant chain where the respondent was surveyed, and the survey round.

We ran alternative model specifications replacing the linear measure for minimum distance with (1) a count of the number of food outlets within a network buffer; and (2) a dummy variable for the presence of a food outlet within a network buffer. We separately ran an ordered logistic regression model with our outcome specified as the frequency of SSB consumption in the past 7 days (i.e., 0, 1–2 times per week, 3–4 times per week, 5–6 times per week, once a day, 2–3 times per day, 4–5 times per day, 6 or more per day). We also ran an alternative model with the outcome specified as whether or not the survey respondent reported “Does not buy soda” to the question “Where do you buy regular soda most often?” All regressions were estimated using Stata version 13.

## Results

The sample was split almost equally across gender (51% male), and was predominantly either African American (39%) or Hispanic (37%) (Table 1). Over half of the sample had a high school degree or more (60%) and was employed (65%). Respondents most commonly lived in Manhattan (39%), followed by Brooklyn (26%), the Bronx (21%) and Queens (14%). The mean distance to any SSB vendor was 213 feet (64.9 meters) (SD = 300 feet (91.44 meters)), the nearest vendor was a fast food restaurant (353 feet (107.6 meters)), followed by a corner store (372 feet (113.4 meters)), and finally a supermarket (1290 feet (393.2 meters)). Approximately 14% of geocoded survey respondents reported consuming a SSB at least daily. Additionally, 7% of the sample reported purchasing a SSB at a corner store daily, 7% reported purchasing a SSB at a supermarket daily, and, 4% reported purchasing a SSB at a fast food restaurant daily. Full details regarding daily and weekly SSB consumption by the vendor type at which SSBs were most frequently purchased are available in S1 Table.

**Table 1:** Demographic characteristic of study sample, overall and by exposure specification.

Demographic characteristic	N (%)	Linear measure for nearest store distance (mean (SD))	Count of the number of stores in 264 feet (80.5 meters) (mean (SD))	Presence of a store in 264 feet (80.5 meters)(%)
Total	3,266	212.7 feet (64.8 meters) (299.9 (91.4 m))	12.1 (12.3)	80.7%
Gender				
Male	1,672 51.2%	207.0 (63.4 m) (290.5 (88.5 m))	12.4 (12.3)	81.0%
Female	1,594 48.8%	218.6 (66.6 m) (309.4 (94.3m))	11.7 (12.3)	80.4%
Race				
White	557 17.1%	215.3 (65.6 m) (300.0 (91.4 m))	13.7 (13.8)	78.6
African American / Black	1,257 38.5%	225.2 (68.6 m) (322.2 (98.2 m))	10.3 (10.7)	80.0
Hispanic	1,196 36.6%	189.9 (57.9 m) (253.1 (77.1 m))	12.9 (12.5)	83.0
American Indian, Asian and Other	256 7.8%	252.1 (76.8 m) (376.5 (114.8 m))	13.4 (13.8)	77.7
Age Group				
18–24	680 20.8%	233.8 (331.0)	11.7 (12.5)	77.2%
25–39	1,153 35.3%	195.7 (282.4)	12.6 (12.8)	83.4%
40–49	569 17.4%	222.2 (306.9)	11.9 (12.0)	81.2%
50–64	628 19.2%	214.1 (291.8)	11.5 (11.6)	78.7%
65+	236 7.2%	208.5 (290.6)	12.4 (11.8)	81.4%
Education Level				
More than a high school degree	1,975 60.5%	226.7 (69.1 m) (310.2 (94.5 m))	12.2 (12.8)	78.9%
High school degree or less	1,291 39.5%	191.3 (58.3 m) (282.3 (86.1 m))	11.8 (11.4)	83.4%
Employment Status				
Not employed	1,159 35.5%	199.9 (60.93 m) (268.8 (81.93 m))	11.8 (11.5)	82.1%
Employed	2,107 64.5%	219.7 (67.0 m) (315.6 (96.2 m))	12.2 (12.7)	80.0%
Borough of the Intersection				
Bronx	683 20.9%	221.1 (67.4 m) (256.1 (78.1 m))	9.3 (9.7)	78.0%
Brooklyn	836 25.6%	222.4 (67.8 m) (308.5 (94.0 m))	10.2 (11.3)	78.2%
Manhattan	1,280 39.2%	143.9 (43.9 m) (192.3 (58.6 m))	15.3 (12.5)	89.7%
Queens	467 14.3%	371.8 (113.3 m) (472.8 (114.1 m))	10.8 (14.6)	64.2%

In Table 2 we report results from models estimating the association between distance to nearest SSB vendor and daily consumption of a SSB. There was no statistically significant effect for distance to the closest SSB vendor on the odds of consuming a SSB daily (OR = 0.99, 95% CI: 0.95, 1.02). We also report models that estimate the association between distance to nearest type of SSB vendor and daily SSB purchase from a corner store, fast food restaurant, or supermarket. None of the estimates from these models was statistically significant.

**Table 2:** Regression results predicting the purchase of a SSB once a day, overall and by food outlet type.

	Consumes a SSB once a day	Purchase a SSB once a day at corner store	Purchase a SSB once a day at fast food restaurants	Purchase a SSB once a day at supermarket
**Outcome percentage**	14.1%	7.1%	3.9%	6.9%
**Linear Measure for closest store distance (in 100 feet (30.48 meters))**	0.99	0.98	0.99	0.99
	(0.95,1.02)	(0.95,1.02)	(0.94,1.04)	(0.98,1.01)
**Gender**				
Male	1	1	1	1
Female	0.73**	0.50***	0.72	0.98
	(0.59,0.90)	(0.37,0.68)	(0.48,1.07)	(0.74,1.30)
**Race**				
White	1	1	1	1
African American / Black	0.78	1.10	0.35**	0.75
	(0.56,1.09)	(0.67,1.82)	(0.18,0.68)	(0.48,1.16)
Hispanic	0.89	1.08	0.93	0.78
	(0.63,1.24)	(0.65,1.79)	(0.52,1.68)	(0.50,1.21)
American Indian, Asian and Other	0.95	0.93	1.24	0.85
	(0.59,1.51)	(0.46,1.86)	(0.58,2.66)	(0.46,1.59)
**Age Group**				
18–24	1	1	1	1
25–39	0.85	0.63**	0.82	0.92
	(0.64,1.12)	(0.44,0.89)	(0.50,1.35)	(0.63,1.35)
40–49	0.92	0.58**	0.90	1.23
	(0.67,1.26)	(0.38,0.87)	(0.51,1.60)	(0.81,1.87)
50–64	0.58**	0.20***	0.57+	0.82
	(0.41,0.81)	(0.12,0.34)	(0.31,1.06)	(0.53,1.27)
65+	0.25***	0.10***	0.23*	0.32**
	(0.14,0.46)	(0.03,0.28)	(0.07,0.70)	(0.14,0.73)
**Education Level**				
More than a high school degree	1	1	1	1
High school degree or less	1.34*	1.58**	1.03	1.18
	(1.07,1.68)	(1.15,2.16)	(0.67,1.58)	(0.87,1.60)
**Employment Status**				
Not employed	1	1	1	1
Employed	0.78*	0.81	1.04	0.86
	(0.62,0.99)	(0.59,1.12)	(0.68,1.61)	(0.63,1.17)
**Borough of the Intersection**				
Bronx	1	1	1	1
Brooklyn	0.71+	0.69	0.44*	0.65+
	(0.51,1.01)	(0.43,1.10)	(0.23,0.84)	(0.41,1.03)
Manhattan	0.75+	0.70+	0.65+	0.79
	(0.55,1.01)	(0.47,1.06)	(0.40,1.08)	(0.53,1.16)
Queens	0.89	1.04	0.39*	0.83
	(0.60,1.30)	(0.62,1.74)	(0.18,0.84)	(0.49,1.40)

Note: The outcome variable is the purchase or consumption of a SSB at least once a day. A corner store is defined as a food retailer that is less than 2,000 square feet (185.8 square meters). A supermarket is defined as a food retailer that is over than 5,000 square feet (484.5 square meters). In parentheses are 95% confidence intervals. Each column represents a separate multivariate logistic regression, adjusted for covariates. In the table we report the odds ratio for each of the covariates and the confidence intervals in parentheses. Crude results are available in S3 Table.

p<0.10 = +.

p<0.05 = *.

p<0.01 = **.

p<0.001 = ***.

Finally, we estimated an ordered logistic regression where the outcome was specified as the frequency with which the respondent reported purchasing a SSB; and separately, we estimated a logistic model predicting the likelihood of a respondent not purchasing a SSB in the past week. Distance was not a statistically significant predictor in either specification (see S4 Table). We also did not observe statistically significant associations between the count of the number of food outlets within a network buffer and daily consumption of a SSB; nor between the presence of a food outlet within a network buffer and daily consumption of a SSB (S4 and S5 Tables).

## Discussion

In this study of frequency of SSB consumption we did not find a correlation between proximity of SSB vendors and the frequency of self-reported SSB consumption among consumers who frequent fast food restaurants. Our results also indicate that distance to the nearest SSB vendor does not influence SSB consumption frequency, regardless of the type of vendor. These results are consistent with a similar study, which did not find a correlation between distance to SSB vendors and the frequency of SSB consumption in Los Angeles, California, another dense urban area.[11]

Our findings may be due to the active transportation environment in NYC, which enables consumers to access SSB vendors easily, including vendors that are far away from their homes. This high transportation access environment may mean respondents do not need SSB vendors near their homes in order to access SSB vendors. Finally, our survey did not include questions about diet soda, which may have replaced SSBs for some consumers.

Our results indicate that fast food consumers in dense urban areas who purchase and consume soda may be resilient to differences in SSB vendor proximity. With this in mind, limiting availability and access to SSB vendors may not be an effective way to curb SSB consumption for this population. Related to this finding, a recent policy intervention known as the NYC Portion Cap Rule aimed to reduce SSB access and availability by limiting the size of SSBs sold in NYC. The policy was met with substantial political and legal resistance that prevented its passage into law, providing further evidence that interventions designed to limit SSB availability and access in dense urban areas may not be feasible.[12]

Food environment interventions should instead target other dimensions of the food environment. For example, SSB-taxes that affect SSB affordability are gaining popularity and have shown promise with respect to their ability to curb SSB consumption, even in cities.[13–15] Though these initiatives require substantial political will, they may be more feasible and effective than initiatives that limit SSB availability and accessibility. Additionally, further research should be conducted in rural areas and areas that better resemble “food deserts”, where the relationship between the food environment and SSB purchase and consumption may differ compared to dense urban areas.

Our study has several limitations. We used a point of purchase sample of customers at fast food restaurants, and we failed to collect a response rate for the survey, though a similar study reports a rate of 60%.[16] Only 56% of cross-streets could be geocoded due to problems with data quality, including misspelled or inaccurate responses to the cross-street item. These misspellings and inaccuracies made it impossible for geocoding software to identify respondents’ cross-streets. Respondents who could not be geocoded were more likely to have a high school degree or less, more likely to be African American, and more likely to be employed; thus, our results may not be generalizable to all fast food consumers. While we use verified self-reported measures for soda consumption, these are not measures of actual consumption, and one-item screeners may be biased. The short-survey format did not allow sufficient time for 24-hour dietary recalls nor prospective SSB consumption measurements. Our study assumed that all food vendors in NYC sell SSBs. Though we could not find research that characterizes the entire NYC SSB environment, anecdotally we have not encountered a store where this is not true.

Finally, though we expected our sample to consume SSBs more frequently than average, instead we found low SSB consumption among our sample; according to the 2013 NYC Department of Health and Mental Hygiene Community Health Survey, 23.3% of NYC residents consumed an SSB daily in 2013, compared to 14% in our sample.[3] The Community Health Survey may provide a better estimate of SSB consumption among all NYC residents because it utilizes a representative sample, which suggests that our results may not be generalizable to all SSB consumers in NYC. Further, our results may not be generalizable outside of urban areas with dense food environments.

This study also has important strengths. First, it utilizes a comprehensive database of food vendors in NYC; though validity and reliability of these data are absent from the literature, we are confident that these data capture a virtual census of NYC food retail outlets open at any given time. Second, it builds on the current literature, replicating null findings from a related study. Finally, it studies a population that is often invoked when considering policy tools to address SSB consumption, and, in combination with previous literature, can be used to inform obesity policy.

## Conclusions

Our findings suggest that neither distance to different types of SSB vendors, presence of SSB vendors, nor total number of SSB vendors have a substantial impact on SSB consumption. Based on these results we conclude that limiting access and availability is likely not an effective solution to decrease SSB consumption among those who frequent fast food restaurants in dense urban areas. Alternatively, policymakers and public health practitioners may need to focus on promoting healthy food purchases and consumption within food stores and restaurants, potentially via interventions such as the Healthy Bodegas initiative, or taxes on SSBs.

## Supporting information

S1 Table Frequency of SSB consumption by most frequent purchase location. (XLSX)

S2 Table Demographic characteristics by geocoded status. (XLSX)

S3 Table Crude regression results predicting the purchase of a SSB once a day, overall and by food outlet type. (XLSX)

S4 Table Predicted frequency of soda consumption once a day with dummy variable for distance buffers around respondents’ home intersections. (XLSX)

S5 Table Predicted frequency of soda consumption once a day with buffer counts. (XLSX)
